# Expression of membrane Hsp90 is a molecular signature of T cell activation

**DOI:** 10.1038/s41598-022-22788-8

**Published:** 2022-10-27

**Authors:** Scott A. Scarneo, Aaron P. Smith, Jacob Favret, Robert O’Connell, Joy Pickeral, Kelly W. Yang, Guido Ferrari, David R. Loiselle, Philip F. Hughes, Manjusha M. Kulkarni, Madhusudhana Gargesha, Bryan Scott, Debashish Roy, Barton F. Haynes, Jesse J. Kwiek, Timothy A. J. Haystead

**Affiliations:** 1 Department of Pharmacology and Cancer Biology, Duke University School of Medicine, 308 Research Drive, Durham, NC 27710 USA; 2 Bolder BioPath INC., Boulder, CO 80301 USA; 3 Department of Surgery, Duke University School of Medicine, Durham, NC 27710 USA; 4 Department of Microbiology, Ohio State University, Columbus, OH 43210 USA; 5 BioInVision, Inc., Mayfield, OH 44143 USA; 6 Department of Medicine, Duke University School of Medicine, Durham, NC 27710 USA

**Keywords:** Biomarkers, Diagnostics, Medicinal chemistry, Pharmacology, Target identification, Target validation

## Abstract

Heat shock protein 90 (Hsp90) maintains cellular proteostasis during stress and has been under investigation as a therapeutic target in cancer for over two decades. We and others have identified a membrane expressed form of Hsp90 (mHsp90) that previously appeared to be restricted to rapidly proliferating cells exhibiting a metastatic phenotype. Here, we used HS-131, a fluor-tethered mHsp90 inhibitor, to quantify the effect of T cell activation on the expression of mHsp90 in human and mouse T cells. In cell-based assays, stimulation of human T cells induced a 20-fold increase in mHsp90 expression at the plasma membrane, suggesting trafficking of mHsp90 is regulated by TCR and inflammatory mediated signaling. Following injection of HS-131 in mouse models of human rheumatoid arthritis and inflammatory bowel disease, we detected localization of the probe at sites of active disease, consistent with immune cell invasion. Moreover, despite rapid hepatobiliary clearance, HS-131 demonstrated efficacy in reducing the mean clinical score in the CIA arthritis model. Our results suggest mHsp90 expression on T cells is a molecular marker of T cell activation and potentially a therapeutic target for chronic diseases such as rheumatoid arthritis.

## Introduction

T cells play an integral role in the host adaptive immune response, choreographing immune surveillance and the host response to infection^1^. Following T cell receptor (TCR) stimulation, T cells undergo rapid activation and replication, and respond to their target antigens by secreting immunoregulatory cytokines and chemokines or through direct cell killing^2^. These rapid cellular changes are governed by TCR mediated protein kinase signal transduction, which increases the expression of specific CD markers essential to the T cell response^3,4^. Heat shock protein 90 (Hsp90) and some of its co-chaperones are upregulated during T cell activation and have been shown to play an important role in regulating many protein kinases essential for T cell activation^5–7^. Hsp90 inhibition has also been shown to block T cell function in vivo, as seen in mouse models of allograft rejection and in rheumatoid arthritis^8^. Thus, Hsp90’s role in mediating T cell proliferation is essential to T-cell mediated immune surveillance.

Recently, we and others characterized a membrane bound form of Hsp90 (mHsp90) that was upregulated on the surface of rapidly proliferating tumor cells exhibiting a metastatic phenotype^9–13^. This phenomenon was originally characterized by Neckers and colleagues, showing that antibodies to Hsp90 block cell migration in tumor lines expressing the surface form of the protein^14^. Subsequently, using cell impermeable fluor-tethered inhibitors of Hsp90, we utilized confocal microscopy to study the trafficking of mHsp90 on the surface of various aggressive tumor cell lines^9,11^. These studies revealed that the protein was actively trafficked to the plasma membrane (PM) under oncogene control and upon binding of a tethered Hsp90 inhibitor, formed aggregates (90 bodies) that were actively reinternalized^11^. In vivo studies with tethered Hsp90 inhibitors in mice bearing breast tumors showed expression of mHsp90 was indeed associated with malignant tumor cells^11^. Subsequently, a tethered inhibitor of Hsp90 carrying a far red fluorophore (HS-196) has entered phase 1 clinical trials as a diagnostic of tumor malignancy.

Here, we show that activated CD^69+^/CD^25+^ T cells express high levels of mHsp90 at the plasma membrane (PM) in response to anti-CD3/CD28 antibody challenge, suggesting that trafficking of mHsp90 is a regulated process in T cells and not a phenomenon of cellular stress such as occurs in oncogenic transformation. Expression of the protein in T cell populations was followed both in vitro and in vivo with HS-131, a Cy5 carrying tethered inhibitor of mHsp90. Furthermore, systemic evaluation of mHsp90 expression in murine models of autoimmune disease shows that tissues with robust expression of mHsp90 also show histopathological evidence of active disease. Our results show that targeting of mHsp90 provides a diagnostic means to evaluate active inflammatory diseases sites and suggest that tethered inhibitors of Hsp90 could be developed as a means to achieve precision immunosuppression in vivo.

## Results

### Regulation of mHsp90 chaperone machinery in T cells

**Figure 1:**
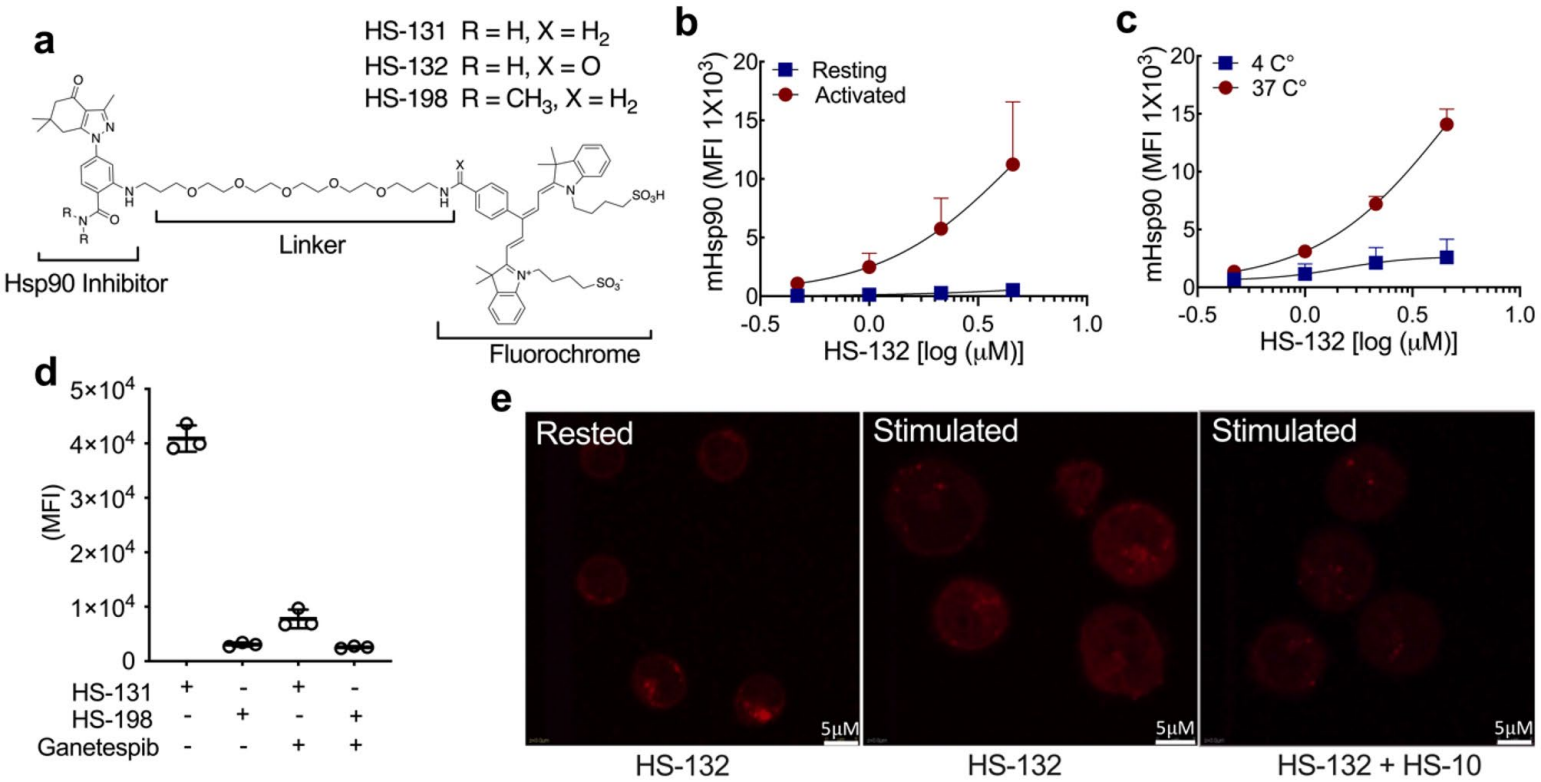
T cell activation induces extracellular Hsp90 (mHsp90) expression. (**a**) Chemical structure of HS-131, and HS-132 active molecule, the inactive analogue HS-198 (negative control). (**b**) Naïve CD3+ T cells isolated from healthy human peripheral blood mononuclear cells (PBMC’s) were activated with anti-CD28 and anti-CD3 antibodies, or resting (unstimulated) for 96 h and treated with HS-132 at the indicated concentrations. Data represented as mean ± SD, 6 biological replicates. (**c**) Internalization of probe by CD3+ T cells at physiological (37 °C) and non-physiological (4 °C) temperatures at varying concentrations of HS-132. Data represents mean ± SD, 4 biological replicates. (**d**) CD3+ T cells isolated from stimulated human PBMC’s were treated with 10 µM HS-131 (active molecule) and HS-198 (inactive control) ± a tenfold excess of ganetespib. Data represents mean ± SD, 3 biological replicates. (**e**) Confocal analysis of HS-132 uptake in stimulated CD3+ T cells, which is blocked with treatment of the non-fluorochrome tethered Hsp90 inhibitor HS-10.

A hallmark of T cell function is to proliferate rapidly in response to antigen presentation along with appropriate cytokine and costimulatory signaling. This leads to the induction of the chaperone machinery which is essential in facilitating this process. In particular, Hsp90 has been shown to be upregulated following T cell immune or cytokine challenge^2,4,5,7^. Therefore, to investigate whether proliferating CD3 + T cells similarly express mHsp90 during T cell activation we examined their ability to absorb fluor-tethered Hsp90 inhibitors following an anti-CD3/CD28 challenge (Fig. 1). Figure 1a shows the structures of two types of fluor-tethered Hsp90 inhibitors used in our studies. The probes consist of an Hsp90-binding ligand, a polyethylene-based tether (linker) and a fluorophore (Cy5 dye ex640 nm/em680 nm). Both the ligand and the fluorophore moieties can be substituted with a variety of small molecules. For example, in our studies we synthesized 3 types of Cy5 probe in which the ligand was either substituted to an N’N dimethylamide to create an inactive control molecule (HS-198) or equally potent but cold-soluble (4 °C) analog by substituting the benzylamine (HS-131) with a benzamide moiety (HS-132). Flow analysis of peripheral blood mononuclear cells (PBMCs) treated with or without CD3/CD28 antibodies showed dose dependent uptake of HS-132 in the activated CD3 + T cell population, in contrast to resting T cells (Fig. 1b, Supplemental Fig. 1a). When the study was repeated at 4 °C versus 37 °C, uptake of the probe was blocked at 4 °C, suggesting that uptake involves active cellular processes rather than simple diffusion (Fig. 1c). Similar temperature dependent effects were observed previously with malignant tumor cell lines^11^. Figure 1d shows the uptake of HS-131 is mHsp90 specific, since minimal uptake was observed with the HS-198 inactive analogue. Additionally, the fluor signal was effectively blocked by competition with a saturating dose of a structurally non-related ATP competitive inhibitor of Hsp90, ganetespib^15,16^. Therefore activated CD3 + T cells internalize fluor-tethered Hsp90 inhibitors due to interactions with the ATP binding site of mHsp90 expressed at the plasma membrane. Confocal analysis of isolated activated human T cells confirms uptake of HS-132 following CD3/28 stimulation compared to resting (inactivated) CD3 + T cell populations (Fig. 1e). Furthermore, the binding of HS-132 was eliminated by HS-10, a non-fluorescent Hsp90 inhibitor. (Fig. 1e).

**Figure 2:**
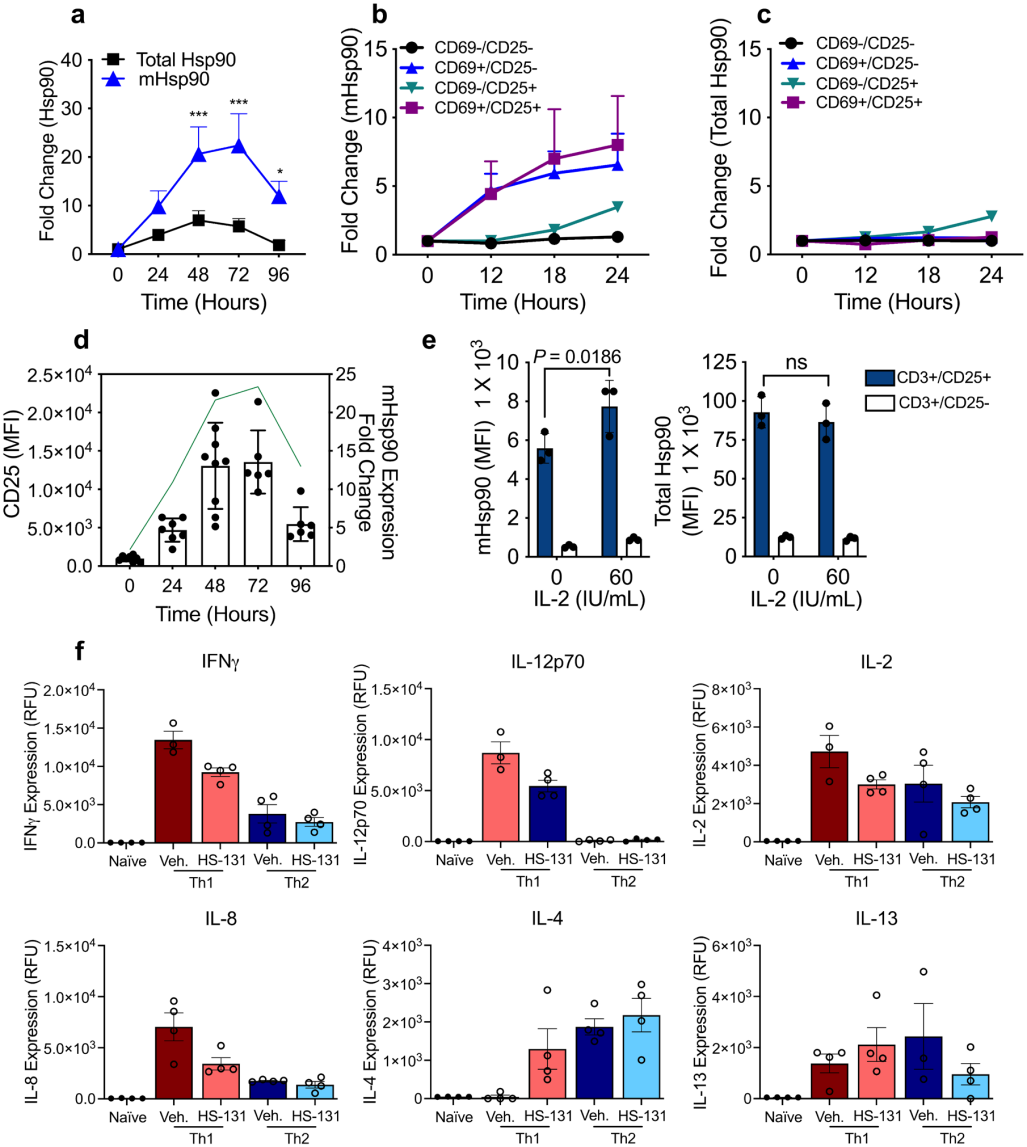
(**a**) Temporal upregulation of mHsp90 compared to total Hsp90 levels in T cells stimulated with CD3/CD28 antibodies over a 96-h period. The data were analyzed by 2-way ANOVA with Sidak’s multiple comparison test, *p < 0.05, **p < 0.01, ***p < 0.001. Experiments were performed with 6 biological replicates. (**b**) Flow cytometric analysis of CD3, CD25, and CD69 T-cell populations relative mHsp90 expression compared to baseline. (**c**) Upregulation of total Hsp90 in activated T cell populations. Experiments were performed with 3 biological replicates for each timepoint (**b**, **c**). (**d**) Mean (± SD) CD25 expression on T cells 24–96 h post CD3/28 stimulation. Temporal upregulation of mHsp90 (green line) post T cell stimulation. CD25 expression; 6–8 biological replicates per time point. mHsp90 expression 6 biological replicates for each timepoint. (**e**) mHsp90 and total Hsp90 expression in CD3+ T cells isolated from PBMC’s were stimulated with IL-2 (60 IU/mL) for 24 h. Data represent mean ± SD. The data were analyzed by 2way ANOVA with Sidaks multiple comparison test. Experiments were performed once with 3 biological replicates. (**f**) Cytokine expression 24 h post Th1 or Th2 T cell stimulation treated with or without HS-131 (1 μM). Data represent mean ± SEM. The data were analyzed by One-way ANOVA with Sidaks multiple comparison test *p < 0.05,**p < 0.01, ***p < 0.001. Experiment represents 3–4 biological replicates per treatment.

We next sought to track the expression of mHsp90 and total Hsp90 over the course of T cell activation. We evaluated both total Hsp90 cellular content and mHsp90 expression in anti-CD3/28 stimulated human PBMCs. Total Hsp90 expression is upregulated 5–7-fold in CD3+ T cells at 2 to 3 days post activation, followed by a reduction in Hsp90 expression. In contrast, mHsp90 expression, as determined by HS-131 binding, was upregulated 20–25-fold following activation (Fig. 2a). Specifically, anti-CD3/28 stimulation caused mHsp90 up regulation in CD69+ /CD25+ and CD69+ /CD25− T-cell populations with a 5–8-fold increase within the first 24 h post activation, whereas CD69−/CD25− T cells show no increase in probe or mHsp90 levels (Fig. 2b). Total Hsp90 was also upregulated in the CD69−/CD25+ populations but only by approximately ~ 2.5 fold (Fig. 2c). When we blocked mHsp90 entry with monoclonal Hsp90 antibodies, no change in viability, the percentage of CD25+ cells or overall cell numbers was observed (Supplemental Fig. 2). This data suggests that mHsp90 may function in the delivery of client proteins to the plasma membrane. One candidate client mHsp90 protein is CD25, the IL-2 receptor. This protein is highly upregulated during T-cell activation due to a positive feedback loop mediated by autocrine and exocrine IL-2^18^. Here we observe that CD25 expression peaks 72 h post activation, followed by a reduction in expression thereafter which correlates with reduced mHsp90 expression (Fig. 2d). Due to the tight correlation of CD25 and mHsp90 expression, we further tested whether exogenous IL-2 stimulation could maintain high expression of both proteins in activated T cells 72–96 h post-stimulation. We found that CD25 expression is significantly upregulated along with mHsp90 in a dose dependent manner in response to IL-2 (*p* = 0.0186) (Fig. 2e). Total cellular Hsp90 expression was not affected by IL-2, suggesting that the trafficking of mHsp90 to the cell surface in T cells is part of a receptor mediated process rather than a reflection of changes in overall Hsp90 protein expression (Fig. 2e). Due to IL-2’s ability to enhance mHsp90 expression in activated cells, we next sought to investigate the impacts of mHsp90 inhibition in activated T cells on inflammatory signaling. Primary human CD3+ T cells were activated with anti-CD3/28 antibodies for 72 h followed by stimulation with either IFNγ/IL-12 (Th1) or IL-4/IL-2 (Th2) for 24 h in the presence of HS-131 (1 μM). Compared to vehicle treated cells, HS-131 significantly reduced IFNγ (*p* = 0.0078), IL-12p70 (*p* = 0.0008), IL-8 (*p* = 0.0057) and significantly increased IL-4 (*p* = 0.048) expression in Th1 stimulated T cells (Fig. 2f). No significant changes were noted between Th2 stimulated and treated cells (Fig. 2f). Furthermore, HS-131 treatment in only anti-CD3/28 stimulated T cells significantly reduced GM-CSF (*p* < 0.001), IFNγ (*p* < 0.001), IL-17A (*p* < 0.001), MIP-1α/β (*p* = 0.013) and TNF α (*p* = 0.025) (Supplemental Fig. 3).

### In vivo tracking of mHsp90 expression in autoimmune disease states

**Figure 3:**
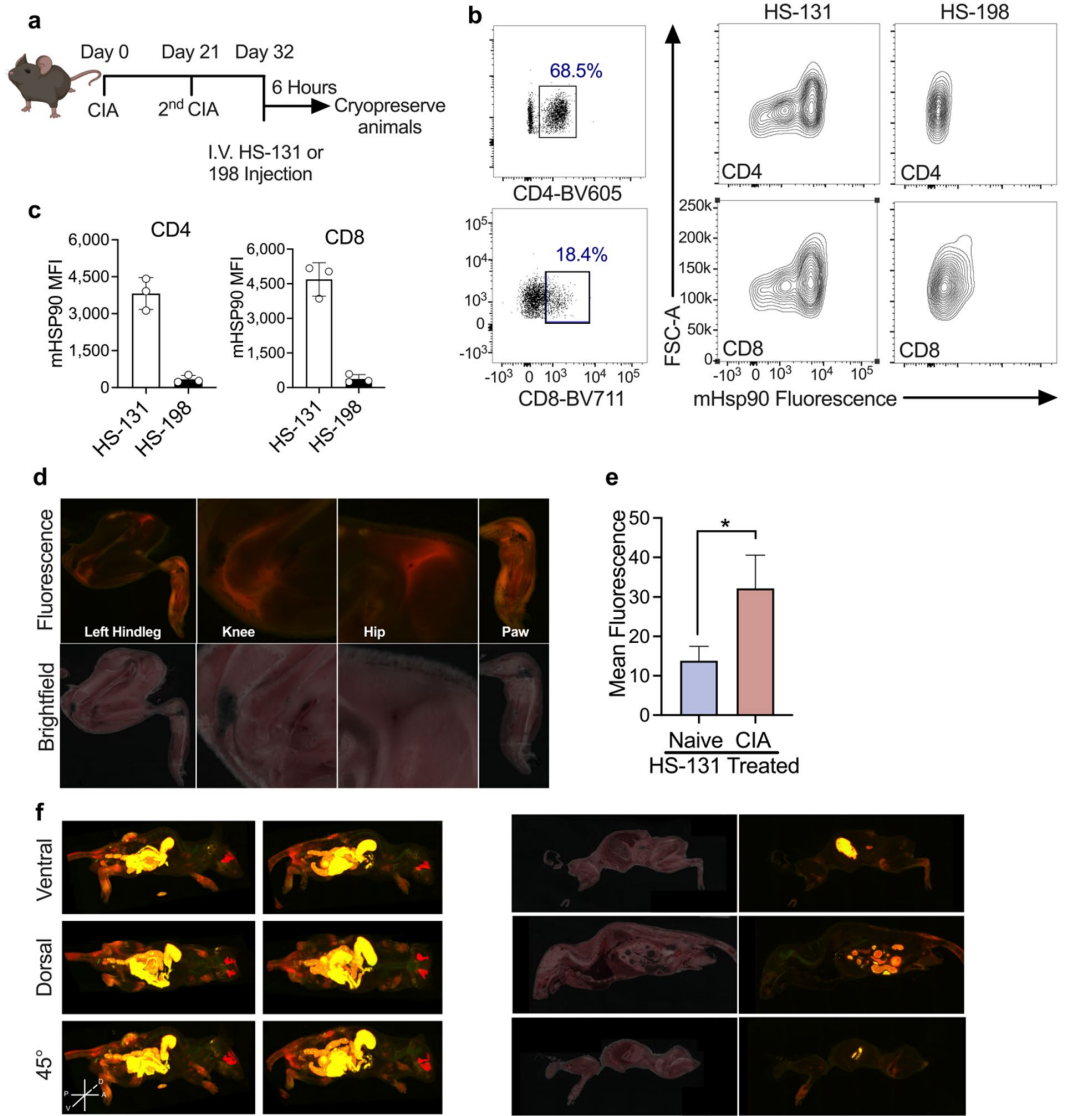
(**a**) Schematic of CIA mouse model of RA experimental design, mice were inoculated with CIA on day 0 and 21. At peak of disease onset (day 32) animals received I.V. HS-131 or HS-198 (inactive control). 6 -hours post treatment mice were whole body cryopreserved. Created with BioRender.com. (**b**,**c**) Flow cytometric analysis of mHsp90 expression on CD4 and CD8 T Cells isolated from the serum of HS-131 and HS-198 treated mice 6 h post tail vein injection. Gated regions indicate CD4 and CD8 T -Cell populations isolated from individual mice. mHsp90 expression in CD4 and CD8 T cells 6 h post HS-131 or HS-198 treatment. *N* = 3 ± SD. Experiments represent 3 biological replicates (**d**) Brightfield and fluorescent histology of hindleg, knee, hip and paw of CIA mice 6 h post tail vein injection of HS-131. (**e**) Quantification of knee joint HS-131 fluorescence compared to naïve animals N = 3 ± SEM. (**f**)Whole body reconstruction of individual HS-131 treated CIA mice 6 h post tail vein injection. Brightfield and fluorescent cross section of CIA mouse.

Previous work suggested that the expression of mHsp90 was restricted to tumor cells exhibiting a metastatic phenotype. Our studies with isolated human PBMCs now suggest that activated T cells also upregulate mHsp90 in response to pro-inflammatory activation. To determine if this phenomenon occurred within the context of a fully functional immune system we utilized the collagen induced arthritic (CIA) mouse model of human rheumatoid arthritis (RA)^8^. Mice (DBA/1lacJ ~ 8 weeks old) reliably develop polyarthritis when immunized against bovine type II collagen^17,18^. The disease that occurs is usually non-symmetric, and any combination of paws/joints may be affected. Mice were injected via the tail vein with bovine collagen at day 0 and then again 21 days later. This second injection triggered the development of the CIA related symptoms over the next several days within the joints (Fig. 3a). A primary mediator of local inflammatory responses to bovine type II collagen are antigen presenting cells (APC’s) followed by invading T cell populations into the joints of the CIA animals. To determine if sites of active disease expressed mHsp90, we injected the animals with HS-131 or HS-198. This was done at the peak of RA symptoms following the second immune challenge. 6-h post HS-131 treatment (i.v.), the animals were euthanized, immediately cryo-frozen in liquid nitrogen and cryo-sectioned (Fig. 3a). Approximately, 500 40 µm slices were made and each slice imaged with both bright field and by fluorescence at Ex640 nm/Em680 nm. Evaluation of the biodistribution was then made by histological examination of individual slices and after in silico reconstruction of the entire mouse anatomy (Fig. 3 and Supplemental movie 1).

T cell phenotyping by flow cytometry of PBMC populations from the CIA mice showed pronounced mHsp90 expression across T-cells as measured by uptake of HS-131. For example, lymphocyte populations of inflammatory CD4 and CD8 cells both showed null, medium and high mHsp90 expression indicative of varying activation states in vivo (Fig. 3b, Supplemental Fig. 4). Minimal signal was obtained after flow studies on PBMCs isolated from CIA mice treated with HS-198, further showing the specificity of HS-131 for mHsp90 in vivo (Fig. 3c). Histological examination of joint slices taken from the cryo-sectioned CIA HS-131 mouse showed enhanced fluorescence in the joints of the animals, which corresponded with peak joint edema observed in the CIA mouse model. In particular, the hind leg, knee, hip and paw all showed a marked increase in HS-131 localization, congruent with infiltration of significant lymphocyte and leukocyte populations (Fig. 3d). Analysis of the whole-body reconstruction of CIA mice shows systemic HS-131 distribution is largely confined to areas of active immune cell invasion such as the hind paws and joints of the animals (Supplemental movie 1). Comparison of HS-131 uptake in a naïve non diseased animal shows no marked HS-131 in the joints including knee when compared to a CIA diseased animal (Fig. 3e, Supplemental Fig. 5a). The movie and sections shown in Fig. 3d,f also highlight the major route of HS-131 elimination which is via the hepatobiliary system and intestines, similar to our prior work. Additionally, the fluorescence associated with the eye shown in Fig. 3 was also noted in previous studies^11,19^. This is a natural fluorescence at Ex640nm/Em680nm associated with the Harderian glands found in rodents^20^. Importantly, no fluorescence was observed in the joints or paws of the tumor bearing animals in our prior studies^11^.

**Figure 4:**
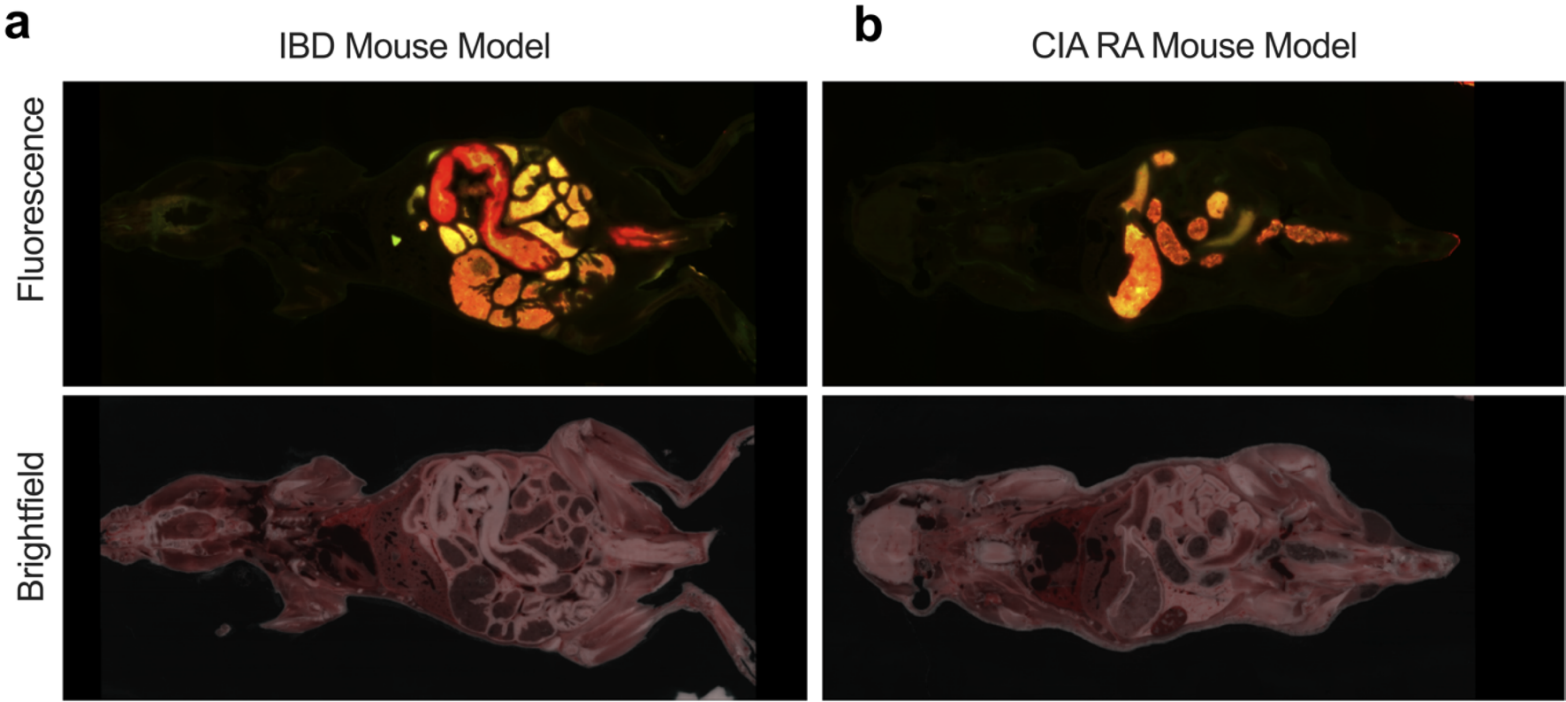
(**a**) mHsp90 expression in an inflammatory bowel disease (IBD) model of auto immune disease. We utilized the T cell transfer model of IBD to evaluate the imaging potential of HS-131 for other autoimmune diseases. Naïve T cells isolated from female donor mice are transferred into SCID mice via IP injection. 3–5 weeks post transfer mice develop inflammation, an extended colon and epithelial hyperplasia. 24 h post HS-131 tail vein injection mice were cryopreserved and imaged as previously mentioned. (**b**) Comparison to CIA mouse 24 h post HS-131 injection. Experiments represent one mouse.

To further illustrate the tissue specificity of HS-131 for sites of active immune cell localization we repeated our cryo-sectioning studies in a mouse model of inflammatory bowel disease (IBD). In this model naïve T cells are isolated and transferred to SCID mice via intraperitoneal injection. Following T cell adoption, the mice develop symptoms of IBD similar to human disease including epithelial hyperplasia, extensive immune cell infiltration and distended colon^21^. Since HS-131 is eliminated through the biliary system and intestine we waited 24 h post HS-131 tail vein injection to image mice in an effort to reduce the non-specific intestinal fluorescence signal associated with hepatobiliary probe clearance. Brightfield imaging of IBD mice showed extensive intestinal distention and inflammation consistent with the development of disease. Similarly, extensive fluorescent signaling from the inflamed bowels indicated areas of high mHsp90 expression consistent with infiltrating immune cell populations (Fig. 4a). In comparison, CIA mice 24 h post HS-131 injection showed no discrete fluorescence associated with the lower intestine (Fig. 4b). Comparison of the biodistribution of HS-131 between the two auto-immune models also illustrated the specificity of the probe for sites of immune induced inflammation. In the case of the IBD mouse, no uptake of HS-131 was observed in any of the animal’s joints or paws (Fig. 4a and b).

**Figure 5:**
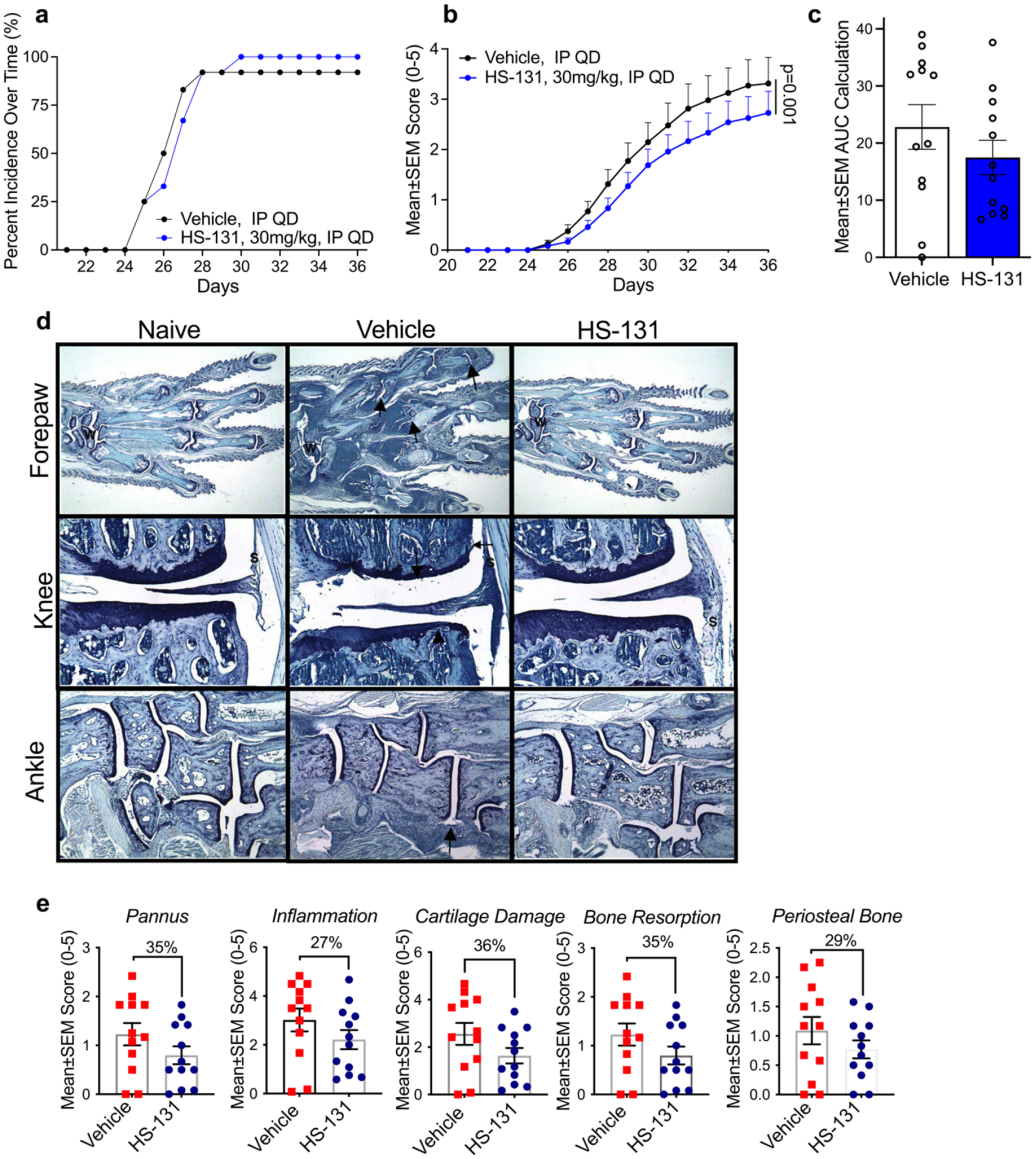
Therapeutic potential of mHsp90 targeted therapies for the treatment of auto immune diseases. (**a**) Animals developing disease, identified by inflammation within at least one paw (**b**) Mean arthritic clinical score of HS-131 30 mg/kg IP, QD and vehicle IP, QD treated mice throughout the study duration *N* = 12/group ± SEM. The data were analyzed by 2way ANOVA with Sidaks multiple comparison test. (**c**) Area under the curve of HS-131 treated mice compared to vehicle N = 12/group ± SEM. (**d**) Representative photomicrographs of forepaw, knee and ankle from a disease control (Naïve), diseased vehicle treated and HS-131 treated animals. W identifies wrist, Arrows identify representative affected joints, (S) identifies inflammation. (**e**) HS-131 reduced pannus, inflammation, cartilage damage, bone resorption and periosteal bone histological manifestations of CIA 36 days post disease onset *N* = 12/group ± SEM. Experiment was performed once with 12 biological replicates per group. Statistical analysis and % inhibition analyzed by unpaired students T-test.

Previous studies have shown that non-tethered Hsp90 inhibitors may act as anti-inflammatory agents by suppressing pro-inflammatory cytokine production in stimulated immune cells^8^. When administered to animals either orally or by injection, these small diffusible molecules are absorbed in all cell types systemically. In contrast, as shown herein and in prior work with tumor disease models, fluor-tethered inhibitors preferentially accumulate in cells expressing mHsp90. Therefore, to test the therapeutic potential of selectively targeting only mHsp90 as an immune-suppressant we tested HS-131 for therapeutic efficacy in the CIA mouse model. First, we established the in vivo bioavailability and pharmacokinetics of HS-131 following i.p. or p.o. administration. Upon daily dosing, following administration of the second collagen challenge we observed that the pathogenesis of disease development in the HS-131 treated group was comparable to the vehicle control (Fig. 5a). However, overall, treatment with HS-131 significantly reduced the mean clinical arthritic score of mice throughout the study period when compared to vehicle control (*p* < 0.001) (Fig. 5b). Analysis of the area under the curve showed a ~ 23% decrease in clinical score between the groups (Fig. 5c). Given the rapid clearance of fluor-tethered versions of Hsp90 inhibitors via the hepato-biliary system, it is remarkable that we observed any efficacy in the CIA model. Histopathologic effects of HS-131 were evaluated in the joints (knee, forepaw, ankle) of disease animals at the study terminus. Disease control animals had histopathologic changes consistent with those seen in type II collagen induced arthritis with microscopic alterations of synovium and periarticular tissue with neutrophils and mononuclear inflammatory cells (inflammation), marginal zone pannus and bone resorption and cartilage damage (chondrocyte death, proteoglycan loss and collagen matrix damage) (Fig. 5d). Histological scoring of the major joints effected by the CIA model showed HS-131 reduced the pannus (35%), inflammation (27%), cartilage damage (36%), bone resorption (35%) and periosteal bone (29%) compared to vehicle treated animals (Fig. 5e). In addition to IP dosing of HS-131, oral dosing showed similar efficacy with ~ 23% reduction in CIA disease score throughout the 36-day study (Supplemental Fig. 6a–c). Our findings suggest that development of longer acting tethered inhibitors of Hsp90 is warranted to achieve a better therapeutic outcome. The promise of such drugs is that they would preferentially target activated immune cells expressing mHsp90 with potentially minimal side effects, in contrast to current broadly acting RA therapies such as TNF-α inhibitors^22–24^.

## Discussion

Here we report that mHsp90 expression is significantly upregulated in activated T cell populations in response to stimulation. Thus, with respect to expression of mHsp90, activated T cell populations behave similarly to tumor cells exhibiting a malignant phenotype^10,11^. By contrast, even in a highly stressed systemic disease state, such as mouse models of RA and IBD, quiescent or fully differentiated cells do not appear to express mHsp90 or absorb HS-131. In malignancy, the precise molecular mechanism governing expression of mHsp90 has yet to be defined. Our studies with IL-2 now demonstrate for the first time that mHsp90 trafficking can be actively regulated through receptor mediated signaling. This process is not merely the consequence of overexpressed Hsp90 due to a general cellular stress response. A possible role for mHsp90 in T cells is the chaperoning of CD surface proteins like CD3, CD4 and CD8, since we and others have noted loss of trafficking of these proteins to the cell surface in the presence of diffusible Hsp90 inhibitors^25^. Additionally, our in vivo work in animal models of autoimmune disease has shown that HS-131 is able to label sites of edema and inflammation, which potentially provides a new way to identify and monitor active inflammatory disease.

We further tested the potential of HS-131 to reduce disease activity in autoimmune diseases. Despite rapid biliary clearance, a small but statistically significant reduction and delay of symptoms was observed in the CIA mouse model of human RA. These findings suggest that longer acting mHsp90 targeted inhibitors could be effective and precise anti-arthritic drugs. In this context, HS-131 may target mHsp90 expression at site of increased inflammation such as the joints of RA patients. Our studies with fluor-tethered inhibitors of Hsp90, first in cancer and now immune disease models, has shown that expression of mHsp90, is associated with rapidly proliferating/activated cells and not fully differentiated cell populations or those preprogramed to be actively replaced such as gut epithelium.

T cells are essential to our immune system’s ability to fight infections; however, many diseases confer maladaptive T cell activation including RA, lupus and IBS as well as other autoimmune diseases. If our hypothesis is correct, the association of mHsp90 expression with T cell activation opens up several avenues that have both diagnostic and therapeutic relevance with respect to autoimmune disease, such as RA. Ultimately our results suggest that further evaluation of the therapeutic potential of mHsp90 inhibitors in autoimmune disease is warranted. patient would not be expected to suffer from long-term immunosuppression.

## Methods

### T-cell activation

PBMC aliquots (ZenBio SER-PBMC-F) were washed in RPMI-1640 with 10% FBS and PSG and then rested overnight in a T-75 flask. The suspended cells were then transferred to a fresh flask and activated in fresh RPMI-1640/10% FBS/PSG by 3:20,000 anti-CD3 (BioLegend 317303), 3:20,000 anti-CD28 (BioLegend 302913), and 1:10,000 (30 IU/mL) human IL-2 (CST 8907SC) and incubated for up to 72 h. In the experiments with longer activation, samples were diluted 1:3 into fresh media containing hIL-2 at 72 h post initial stimulation with CD3/CD28 antibodies. Rested cells were treated similarly except they did not receive activating antibody or hIL-2. Fold change of mHsp90 and Hsp90 expression (Fig. 2) was calculated by comparison of MFI at a specified timepoint compared to expression of mHsp90 or Hsp90 of that same sample at time = 0 h. ${\text{Fold}}\;{\text{Change}} = \frac{{{\text{MFI }}\;{\text{at}}\;{\text{ time}}\;{ }\left( {X{\text{h}}} \right)}}{{{\text{MFI }}\;{\text{at }}\;{\text{time}} \;\left( {0{\text{h}}} \right)}}$.

### Cytokine experiments

Human PBMC aliquots from 4 unique donors were used to isolate T cells as previously described. Isolated T cells were activated with anti-CD3/28 antibodies for 72 h followed by stimulation with Th1: IL-2 (2 ng/mL), IL-12(10 ng/mL) or Th2: IL-4 (10 ng/mL) human cytokines for 24 h.

### Activation inhibition experiment- Hsp90 antibodies

ADI-SPS-771, 9D2, and AC88 (all Enzo) were cleaned with 10kD MWCO spin filters. Cleaned antibody was added in a titration to newly activated or resting PBMCs. The cells were incubated for 72 h and then stained for flow cytometry.

### Western blot

Samples were prepared as described in the specific experiment and then loaded on Criterion 4–15% Tris–HCl gels (BioRad 5678083 and 5678084) and separated at 200 V. The proteins were transferred from the gel to a PVDF membrane (BioRad 1620177) on ice for 1 h at 100 V. After transfer, the membranes were rinsed in 1 × TBST and then blocked for 30 min with either 5% NFDM/TBST or 5% BSA/TBST depending on the block being used for the primary antibody staining step. After blocking, the membranes were labelled with primary by overnight incubation with the manufacturer recommended concentration and blocking agent for each antibody (Table 1). Residual primary antibody was removed by 5-min washes with TBST (3×) and then the appropriate secondary antibody (CST 7074 and 7076) was added at 1:10,000 in 5% NFDM/TBST for 1 h at room temperature. Upon completion of secondary stain, residual secondary antibody was removed by 5-min washes with TBST (3x). Clarity ECL (Biorad 1,705,060) reagent was used to develop film.

**Table 1:** Western blot antibodies.

Antibody	Target	Manufacturer	Buffer	Concentration	Secondary
PA5-27,596	Trap1	Thermo Fisher	5% NFDM	1:1000	Anti-rabbit
ab3280	β-Actin	Abcam	5% BSA	1:5000	Anti-mouse
H-114	Hsp90α/β	Santa Cruz	5% NFDM	1:200	Anti-rabbit
ab56721	Aha1	Abcam	5% BSA	1:1000	Anti-rabbit
5670	Hop	Cell signaling	5% BSA	1:1000	Anti-rabbit
2080	Chip	Cell signaling	5% BSA	1:1000	Anti-rabbit
11826 s	FKBP4	Cell signaling	5% BSA	1:1000	Anti-rabbit
12,210	FKBP5	Cell signaling	5% NFDM	1:1000	Anti-rabbit
D4I5T	Tyk2	Cell signaling	5% BSA	1:1000	Anti-rabbit
5174S	GAPDH	Cell signaling	5% NFDM	1:1000	Anti-Rabbit

### Flow cytometry

The activated/rested cells were plated into a 96-well round bottom plate and pelleted at low speed. Cells were resuspended in chilled media containing titrated HS-131 or HS-198 (in 1% DMSO) and stained for 30 min at RT. Fluorochrome tagged inhibitors and media were washed away with a PBS wash, followed by a PBS wash containing 3% NMS. Surface antibody stains were performed in 3% NMS/PBS for 30 min followed by PBS washes (2×) (Table 2). Samples were analyzed on BD FACSCanto II flow cytometer. Permeabilized samples were prepared as previously described followed by fixation in 4% Formaldehyde/PBS overnight at 4 °C. Minimum gating of 2X10^5^ cells/sample. Sample analysis was performed with Flowing Software and FlowJo.

**Table 2:** Flow cytometry antibodies.

Antibody	Target and fluor	Manufacturer	Concentration
300434	CD3- BV421	BioLegend	1:200
302616	CD25-AF488	BioLegend	1:200
423113	Viability-BV421	BioLegend	1:1000
423102	Viability-BV510	BioLegend	1:1000
310903	CD69-FITC	BioLegend	1:200
302605	CD25-PE	BioLegend	1:200
561105	Ly6G-AF488	BD Biosciences	1:500
103139	CD45-BV605	BioLegend	1:800
563415	IA/IE-BV650	BD Biosciences	1:1500
103121	CD45-AF488	BioLegend	1:1000
300454	CD3-AF488	BioLegend	1:1000
301817	CD14-AF488	BioLegend	1:1000
400625	Rat IgG2b Isotype Control	BioLegend	1:1000

### CIA disease

CIA induction was performed as previously described^17^. Collagen was prepared at 4 mg/ml in 0.01 N acetic acid. Equal volumes of 4 mg/ml collagen and 5 mg/ml Freund’s complete adjuvant were emulsified by hand mixing with syringes for approximately 5 min, at which point a bead of this material holds its form when placed in water. On study days 0 and 21, animals were anesthetized with isoflurane and given intradermal injections of a total of 400 μg of type II collagen in Freund’s complete adjuvant at the base of the tail.

### CIA experimental design

All animal studies were approved and performed by Bolder BioPath Institutional Animal Care Use Committee (IACUC) and was in accordance with the National Institutes of Health (NIH) Guide for the Care and Use of laboratory animals. Additionally, all animal studies conformed with the NIH ARRIVE guidelines. 24 DBA/1 mice 12/group were used for these studies. Mice were randomized into treatment groups by body weight on study day 18. Animals were treated from day 21–36 of the study with either 20 μL of HS-131 (30 mg/kg) PO, QD, vehicle (DMSO) PO, QD, HS-131 (30 mg/kg) IP, QD or vehicle (DMSO) IP, QD. On study day 36, the mice were euthanized for necropsy. Clinical scoring system was followed as previously described^17^. Clinical scores were given for each of the paws (right front, left front, right rear, left rear) on study days 21–36. 0 = normal, 1 = one hind or fore paw joints affected or minimal diffuse erythema and swelling, 2 = two hind or fore paw joints affected or moderate diffuse erythema and swelling, 3 = three hind or fore paw joints affected or moderate diffuse erythema and swelling, 4 = four hind or fore paw joints affected or marked diffuse erythema and swelling, 5 = entire paw affected, severe diffuse erythema and severe swelling, unable to flex digits. Experimenter was blinded from the treatment group during clinical evaluation and scoring.

### IBD disease

On study day 0, Balb/C mice were terminated and spleens obtained for CD4 + CD45RB^high^ cell isolation. After cells had been sorted and obtained, each animal received an IP injection of at a minimum 4X10^5^ cells (200 µl/mouse injections). On study day 49, a diseased mouse was treated IV with 20 μl of inflammation tracer HS-131 (10nmoles). One animal was used for this study. The animal was observed for 24 h.On study Day 50, the mouse was euthanized via CO_2_ inhalation, frozen in liquid nitrogen using a black cryoprotectant gel (embedding medium), and sent to BioInVision for analysis.

### CryoViz imaging

Whole mice were imaged at 10.23 μm × 10.23 μm in-plane resolution using an Olympus MVX-10 microscope with a 1X objective and 0.63X magnification and 40 μm section thickness, using the CryoViz (BioInVision, Inc., Cleveland, USA). CryoViz is a fully‐automated, serial sectioning‐and‐imaging system which provides 3‐dimensional, tiled, microscopic anatomical bright field and molecular fluorescence images over large fields-of-view such as a whole mouse. Images were acquired using a dual band FITC/TxRed fluorescence filter (Chroma, Inc., Rockingham, VT), a liquid crystal RGB filter and a low-noise monochrome camera. Raw images acquired by the CryoViz were processed to generate 3D color anatomical brightfield and molecular fluorescence volumes using the CryoViz Preprocessor software (BioInVision, Inc., Cleveland, Ohio). These 3D volumetric image data were then processed using the CryoViz 3D Visualizer software (BioInVision, Inc., Cleveland, USA) to obtain 3D reconstructed brightfield and fluorescence volume renderings and movies with 2D slice cutaway animations, showing the 2D/3D biodistribution of TxRed labeled HS131 within the mouse volume.

### Confocal microscopy

T cells isolated from PBMC’s were plated onto coverslips. The cells were stained with HS-131 and HS-198 at room temperature for 2 h. Following staining, cells were fixed to coverslips with 4% formaldehyde/PBS. The fixative was then removed with PBS washes (3×) and Unbound stain was removed by PBS washes (2×) and then the coverslips were rinsed with ddH2O and affixed to slides with FluorSave (Millipore 345,789). Imaging was performed on a Leica SP5 confocal microscope.

### Immunohistological staining

Fixation of tissues was performed as previously described^17^. Briefly, after 24–48 h in fixative and 4–5 days in 5% formic acid for decalcification, tissues were trimmed, and processed for paraffin embedding. Paws were embedded in paraffin in the frontal plane and the knees were embedded with the patella facing down. Ankles, if left attached to the hind paw, were also embedded in the frontal plane but may be detached and sectioned in the sagittal plane for special purposes. Sections were cut and stained with toluidine blue.

#### Scores for synovitis, pannus formation, degradation of cartilage, and bone

All histological scoring systems were performed as previously described^17^. Sum scores reported are an average of left and right forepaw, left and right hind paw and left and right knee scores of all Histopathology parameters described below on a scale of 0–5.

#### Paw score criteria

0 = Normal. 0.5 = Very minimal, affects only 1 joint or minimal multifocal periarticular infiltration of inflammatory cells. 1 = Minimal infiltration of inflammatory cells in synovium and periarticular tissue of affected joints. 2 = Mild infiltration of inflammatory cells. When referring to paws, generally restricted to affected joints (1–3 affected). 3 = Moderate infiltration with moderate edema. When referring to paws, restricted to affected joints, generally 3–4 joints and the wrist or ankle. 4 = Marked infiltration affecting most areas with marked edema, 1 or 2 unaffected joints may be present. 5 = Severe diffuse infiltration with severe edema affecting all joints (to some extent) and periarticular tissues.

#### Knee score criteria

0 = Normal. 0.5 = Very minimal, affects only one area of the synovium or minimal multifocal periarticular infiltration of inflammatory cells. 1 = Minimal infiltration of inflammatory cells in synovium and periarticular tissue of affected synovial areas. 2 = Mild diffuse infiltration of inflammatory cells. 3 = Moderate diffuse infiltration of inflammatory cells. 4 = Marked diffuse infiltration of inflammatory cells. 5 = Severe diffuse infiltration of inflammatory cells.

#### Cartilage damage score criteria

0 = Normal. 0.5 = Very minimal = Affects marginal zones only of one to several areas (knees) or joints (paws). 1 = Minimal = Generally minimal to mild loss of toluidine blue staining (proteoglycan) with no obvious chondrocyte loss or collagen disruption in affected joints/areas 2 = Mild = Generally mild loss of toluidine blue staining (proteoglycan) with focal areas of chondrocyte loss and/or collagen disruption in some affected joints/areas. Paws may have one or two digit joints with near total to total loss of cartilage. 3 = Moderate = Generally moderate loss of toluidine blue staining (proteoglycan) with multifocal chondrocyte loss and/or collagen disruption in affected joints/areas. Paws may have three or four joints with near total or total loss. In the knee, some matrix remains on any affected surface with areas of severe matrix loss. 4 = Marked = Marked loss of toluidine blue staining (proteoglycan) with multifocal marked (depth to deep zone or tidemark) chondrocyte loss and/or collagen disruption in most joints with a few unaffected or mildly affected. In the knee, one surface with total to near total cartilage loss. 5 = Severe = Severe diffuse loss of toluidine blue staining (proteoglycan) with severe (depth to tide mark) chondrocyte loss and/or collagen disruption in most or all joints.

### Quantification and statistical analysis

Graphpad Prism 8 was used for statistical analysis of T cell activation, mHsp90 expression and viremia. For each analysis, total n and SEM are presented in the figure legend. An alpha of 0.05 was used for all statistical analysis.

## Supplementary Information

Supplementary Information 1.

Supplementary Video 1.

## Data Availability

Data that supports the findings of this study are available from the corresponding author upon reasonable request.
