# Supplementary material for: Expression of membrane Hsp90 is a molecular signature of T cell activation
Source: Sci Rep. 2022 Oct 27;12:18091. doi: 10.1038/s41598-022-22788-8 (PMC9613876; doi:10.1038/s41598-022-22788-8)
Supplement: Supplementary file 1 — Supplementary Information 1. [file 41598_2022_22788_MOESM1_ESM.docx]

**Expression of extracellular Hsp90 is a molecular signature of T cell activation, providing a means to image and target T Cell activation in autoimmune disease**

Scott A. Scarneo^1#^, Aaron P. Smith^1#^, Jacob Favret^2^, Robert O’Connell^2^, Joy Pickeral^3^, Kelly W. Yang^1^, Guido Ferrari^3^, David R. Loiselle^1^, Philip F. Hughes^1^, Manjusha M Kulkarni ^4^, Madhusudhana Gargesha^5^, Bryan Scott^5^, Debashish Roy^5^, Barton F. Haynes^6^, Jesse J. Kwiek^4^ and Timothy A. J. Haystead^1*^

**Supplemental Figures**

**Figure 1s.**

Flow cytometric gating of HS-132 stained CD3+ human T cells. CD3+ T cells were stained at 10μM with or without HS-132.


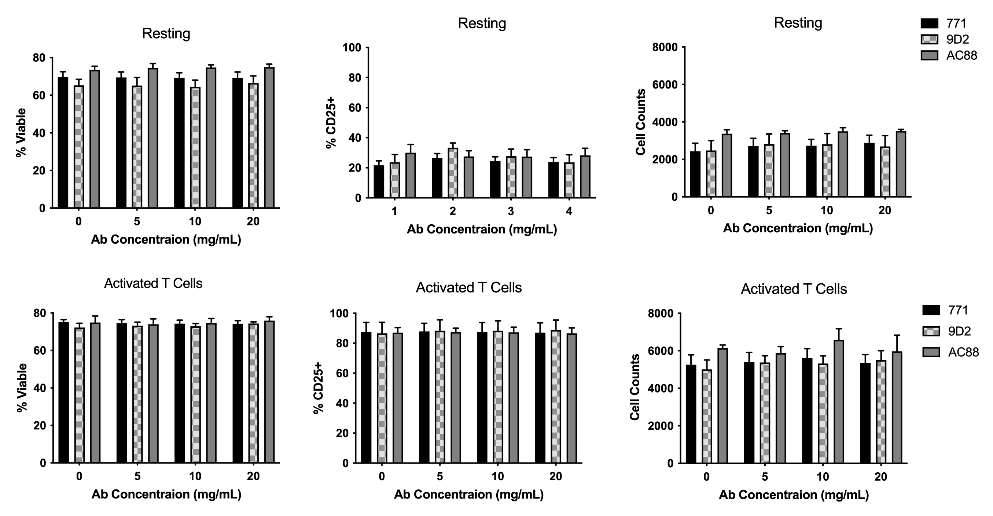


**Figure 2s.**

Effects of three monoclonal Hsp90 targeted antibodies. Hsp90 antibodies were treated at varying concentrations in resting T cells (upper panel) and activated (CD3/28 activation, lower panel). % viability, %CD25 and total cell count recorded. N=3-6 biological replicates/group.

**Figure 3s**

Effects of HS-131 on cytokine expression in CD3/28 T cells. Human CD3+ T cells were treated with HS-131 (1μM) or vehicle followed immediately by stimulation with CD3/28 antibodies. Cell culture supernatants were collected 24-hours post stimulation and cytokines expression determined. Data represent mean­±SEM. The data were analyzed by 2-way ANOVA with Sidaks multiple comparison test *<0.05, ***p<0.001, # represents activated-vehicle treated significantly different from naïve. Experiment 4 biological replicates per treatment.

**Figure 4s.**

Flow cytometric gating scheme for T cell analysis in CIA mice receiving HS-131 or HS-198.


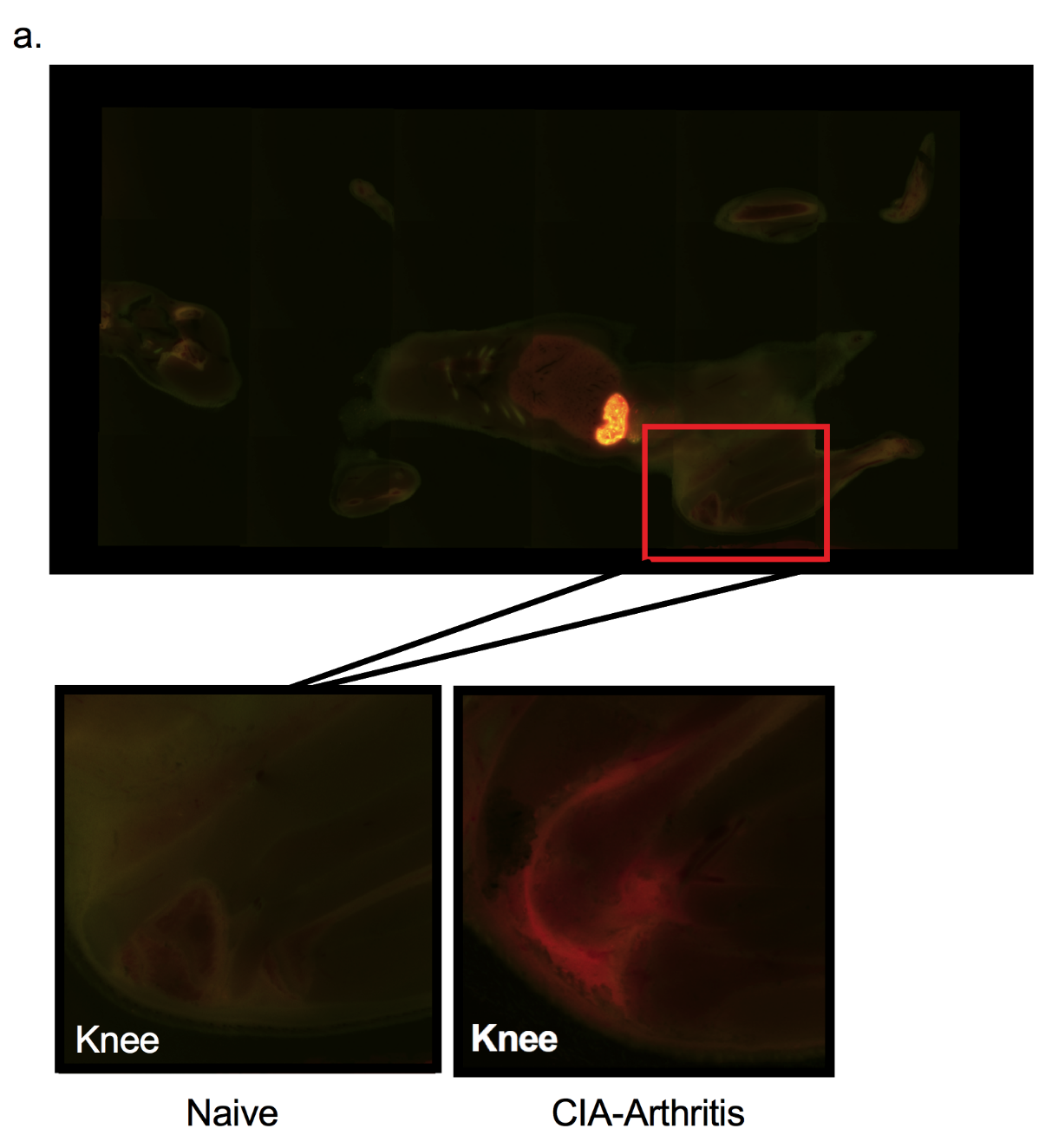


**Figure 5s**

eHsp90 expression in a naïve disease-free mouse injected with HS-131 (10nmoles, tail vein) cryoimaged 6 hours post injection. Comparison of knee joint of naïve mouse and CIA arthritic mouse eHsp90 expression.

**Figure 6s**

Therapeutic potential of eHsp90 targeted therapies for the treatment of auto immune diseases. (A) Disease incidence over time. (B) Mean arthritic clinical score of HS-131 and vehicle treated mice throughout the study duration N=11/group±SEM. (C) Area under the curve of HS-131 treated mice compared to vehicle N=11/group±SEM. Data analyzed by 2-Way ANOVA followed by Sidaks multiple comparisons.

**Supplemental Movie 1**

Reconstruction of whole CIA mouse imaging. At peak of CIA disease mice were injected with i.v. HS-131. 6-hours post injection, mice were sacrificed and cryopreserved prior to slide preparation. Individual mice slices were imaged using a dual band FITC/TxRed fluorescent filter. Reconstruction of 40μm slices of mice in Imaris 9.5 software. CIA
